# A Community-Based System Dynamics Study of the Senior Community Service Employment Program

**DOI:** 10.1093/geroni/igab046.087

**Published:** 2021-12-17

**Authors:** Cal Halvorsen, Kelsey Werner, Elizabeth McColloch

**Affiliations:** Boston College, Chestnut Hill, Massachusetts, United States

## Abstract

The Senior Community Service Employment Program (SCSEP), the only federal workforce-training program that targets older adults, engages people aged 55 years and older with incomes at or below 125% of the federal poverty level with multiple barriers to employment. This study examined SCSEP’s role in participant financial, physical, and mental well-being. To do so, we held five sessions (four virtual, one telephone) over a combined nine hours in August and September 2020 using a form of participatory research called community-based system dynamics with 15 Massachusetts SCSEP participants and case managers. Through structured activities, respondents identified how program, policy, and organizational factors influence and are influenced by participant well-being (e.g., SCSEP participation results in less social isolation, decreased isolation subsequently increases desire to participate) as well as program and policy recommendations to strengthen the program (e.g., reconsider benchmarks of success). These findings highlight the benefits and potential of this long-running program.

